# Efficient Charge Transfer in MAPbI_3_ QDs/TiO_2_ Heterojunctions for High-Performance Solar Cells

**DOI:** 10.3390/nano13071292

**Published:** 2023-04-06

**Authors:** Hua Li, Chao Ding, Dong Liu, Shota Yajima, Kei Takahashi, Shuzi Hayase, Qing Shen

**Affiliations:** 1 Faculty of Informatics and Engineering, The University of Electro-Communications, 1-5-1 Chofugaoka, Chofu, Tokyo 182-8585, Japan; 2 Institute of New Energy and Low-Carbon Technology, Sichuan University, Chengdu 610065, China

**Keywords:** MAPbI_3_, quantum dots, charge transfer, heterojunctions, solar cells

## Abstract

Methylammonium lead iodide (MAPbI_3_) perovskite quantum dots (QDs) have become one of the most promising materials for optoelectronics. Understanding the dynamics of the charge transfer from MAPbI_3_ QDs to the charge transport layer (CTL) is critical for improving the performance of MAPbI_3_ QD photoelectronic devices. However, there is currently less consensus on this. In this study, we used an ultrafast transient absorption (TA) technique to investigate the dynamics of charge transfer from MAPbI_3_ QDs to CTL titanium dioxide (TiO_2_), elucidating the dependence of these kinetics on QD size with an injection rate from 1.6 × 10^10^ to 4.3 × 10^10^ s^−1^. A QD solar cell based on MAPbI_3_/TiO_2_ junctions with a high-power conversion efficiency (PCE) of 11.03% was fabricated, indicating its great potential for application in high-performance solar cells.

## 1. Introduction

Metal halide perovskite semiconductor materials have been extensively investigated for potential applications in photovoltaics (PVs) [1,2], light-emitting diodes (LEDs) [3,4], detectors [5,6,7], and lasers [8,9]. To date, the power conversion efficiency (PCE) of bulk perovskite solar cells (PSCs) has been rapidly improved to 25.7%, which is comparable to that of large-scale commercial crystalline silicon solar cells [10]. Nevertheless, although progress has been achieved over the past few years, some challenges remain unsolved, such as instability resulting from moisture/thermal sensitivity, which hinders commercial applications [11]. The low-dimensional perovskite quantum dots (PQDs) possess new attractive features compared with their bulk counterparts, such as improved defect tolerance [12], enhanced stability [13], high photoluminescence quantum yield (PLQY) [14], and tunable bandgaps over the whole spectrum region [15,16]. More importantly, wide bandgap tunability makes PQDs particularly suitable when acting as a top cell in tandem solar cells [17]. The PQDs with precise size control and high PLQY have been successfully synthesized to date [14,18,19]. Apart from formamidinium lead triiodide (FAPbI_3_) and Cesium lead triiodide (CsPbI_3_ QDs), methylammonium lead triiodide (MAPbI_3_) QDs, as one of the most promising materials has also been used in solar cells due to an appropriate Goldschmidt tolerance factor (*τ* is approximately 0.91) and low-temperature processing preparation [20,21].

To fully comprehend the fundamental photophysical process and take advantages of the unique properties of these QDs in photovoltaic devices, it is generally designed as heterojunction that QDs selectively contact other metal oxides (MOs) such as NiO_x_, TiO_2_ or ZnO, where the MOs work as the charge transport layer (CTL) [19,22,23]. These QD/MO heterojunctions are an integral part of the QD solar cells, in which the main photogenerated charge carriers transfer from QDs to the QD/CTL interface, are extracted by the CTL, and must finally be collected by the electrodes [24]. The dynamic competition between charge transfer and charge recombination is a critical determinant of photovoltaic device performance. Therefore, a deep understanding of the electronic interactions of QDs with the CTL is crucial to enhancing the performance of MAPbI_3_ QD-based optoelectronics. The electron injection rate from bulk MAPbI_3_ to TiO_2_ has been explored in several works [25,26]. However, little is known concerning the photoexcited carrier transfer kinetics at the MAPbI_3_ QDs/TiO_2_ heterojunction. Powerful ultrafast transient absorption (TA) spectroscopy enables us to study charge transfer dynamics [22,27,28]. In this study, we investigated the charge injection dynamics from MAPbI_3_ QDs to TiO_2_ for the first time, and the charge transfer rate of various sizes of QDs was determined and found to increase from 1.6 × 10^10^ to 4.3 × 10^10^ s^−1^ with the average QD size decreasing from 13.3 to 9.4 nm. Finally, A solar cell based on MAPbI_3_/TiO_2_ junctions with a PCE of 11.03% was fabricated, indicating an efficient charge transfer through these junctions.

## 2. Materials and Methods

### 2.1. Materials

Lead (II) iodide (PbI_2_, 99.99%) was purchased from high-purity chemicals. The 9,9′-spirobifluorene (spiro-OMeTAD, ≥99.5%) was purchased from Merck KGaA (Darmstadt, Germany). Methylammonium acetate (MA-acetate, >98%) and formamidinium iodide (FAI, >99.0%) were purchased from Tokyo Chemical Industry Corporation (TCI, Tokyo, Japan). Oleic acid (OA; 90%, technical grade), oleylamine (OAm, 70%, technical grade), 1-Octadecene (ODE, 90%, technical grade) and chlorobenzene (CB, anhydrous, 99.8%), Bis(trifluoromethane)sulfonimide lithium salt (Li-TFSI) and 4-tert-butylpyridine (4-TBP; 96%) were purchased from Sigma-Aldrich (Tokyo, Japan). Hexane (Guaranteed Reagent), methyl acetate (MeOAc, 99.5%, anhydrous), octane (Wako special grade), ethyl acetate (EtOAc, 99.5%, anhydrous) and lead nitrate (Pb (NO_3_)_2_, 99.9%) were purchased from FUJIFILM Wako Pure Chemical Corporation (Osaka, Japan). The 30NR-D titania paste was purchased from Greatcell Solar Materials (Queanbeyan, Australia). Al-Nanoxide A/SP was purchased from Solaronix. All chemicals were used as received.

### 2.2. Colloidal Synthesis of MAPbI_3_ Quantum Dots (QDs)

At first, a 50 mL three-neck flask was charged with 1 g of MA-acetate and 20 mL of OA, and the mixture was dried for 1 min at room temperature (RT) and then heated to 80 °C under nitrogen until all the MA-acetate had reacted with OA, and the MAOA precursor was obtained (it must be preheated to 60 °C before injection). Subsequently, 0.344 g of PbI_2_ and 20 mL of ODE were loaded into a 50 mL three-neck flask and degassed under a vacuum for 1 h at 120 °C. A total of 6 mL of OA and 3 mL of OAm were injected at 120 °C under nitrogen atmosphere. After the complete solubilization of PbI_2_, the mixture was cooled down to 60, 80 or 100 °C, and a 4, 5, or 6 mL MAOA precursor solution was swiftly injected (the volume ratio of MAOA precursor and the injection temperature were regulated to control the QDs size). About 10 s later, the reaction mixture was quenched with an ice-water bath. The crude QD solution was divided into two tubes, and the mixture of 1 mL Toluene together with 5 mL MeOAc was added to each tube, followed by centrifugation at 9300 rpm for 4 min 20 s. The supernatant was discarded, and the precipitate was dried with nitrogen and then dispersed in hexane (the precipitate was dispersed in octane to prepare the MAPbI_3_ QD solar cells). The obtained QD solution was kept in a refrigerator until use.

### 2.3. Fabrication of the QDs/MOs Heterojunction

Mesoporous TiO_2_ and Al_2_O_3_ films were prepared according to our previous reports [19,23]. Briefly, the purchased 30NR-D titania paste was diluted by ethanol and was deposited on a glass substrate via the doctor blade method using mending tape as a spacer before it was then dried at 180 °C for 10 min. Finally, the dried films were calcined in the air at 500 °C for 30 min. The preparation method of the Al_2_O_3_ film was similar to that of TiO_2_. The QDs/MOs heterojunction was obtained by directly adsorbing the QDs on MOs; that is, the mesoporous TiO_2_ or Al_2_O_3_ films were immersed in a MAPbI_3_ QD colloidal solution in hexane for 8 h, subsequently, the adsorbed QD films were rinsed in hexane and dried with nitrogen.

### 2.4. Fabrication of the MAPbI_3_ QD Planar Heterojunction Solar Cells

The FTO glass substrates were cleaned with successive sonication in ethanol, acetone, and 2-propanol for 20 min, respectively, and then treated in an ultraviolet-ozone chamber for 10 min before use. The compact TiO_2_ layer with approximately 50 nm was deposited on FTO substrates and annealed at 450 °C for 30 min [29]. The MAPbI_3_ colloidal QD solution in octane with a concentration of 100 mg/mL was spin-cast on TiO_2_ at 2000 rpm for 20 s in a nitrogen glovebox; the resulting film was dipped into a Pb (NO_3_)_2_ in MeOAc saturated solution for 5 s, rinsed with neat MeOAc 3 times, and dried at 2000 rpm for 20 s. The above QD deposition process was repeated four times to achieve the desired film thickness. After that, the films were soaked in FAI in EtOAc saturated solution for 10 s, and dried at 2000 rpm for 20 s. The spiro-OMeTAD (0.09 g of spiro-OMeTAD, 1 mL of chlorobenzene, 22 μL of Li-TFSI (520 mg/mL) and 36 μL of 4-TBP) was then spin-cast on the QD absorber layers at 5000 rpm 30 s. Finally, the Au electrode with a thickness of 80 nm was deposited using thermal evaporation. The active surface area of the solar cells was 0.16 cm^2^.

### 2.5. Characterization

The morphologies of MAPbI_3_ QDs and MAPbI_3_ QDs/TiO_2_ nanoparticles were obtained using high-resolution transmission electron microscopy (HRTEM, JEM-2100F, Akishima, Japan). The UV-vis absorption spectra for all samples were measured by a spectrophotometer (HITACHI, U-3900H, Tokyo, Japan). The phase identification was performed on a powder X-ray diffraction (XRD, TTR-III, Rigaku Corporation, Tokyo, Japan). The photoluminescence quantum yield (PLQY) of the QD solution was obtained with an integrating sphere of an Absolute PL Quantum Yield Spectrometer system (C11347, Hamamatsu Photonics, Hamamatsu, Japan), and the excitation power was 0.1 mW. The photoelectron yield spectra (PYS) were recorded by an ionization energy measurement system (Model BIP-KV205, Bunkoukeiki Co., Ltd., Tokyo, Japan). Time-resolved PL (TRPL) spectra were characterized by a NIR PL lifetime spectrometer (C12132, Hamamatsu Photonics, Hamamatsu, Japan). The J–V curves measurement of MAPbI_3_ quantum dot solar cell was performed on a Keithley 2400 digital source meter (Tektronix, Tokyo, Japan) under AM 1.5 irradiation at a scan step of 0.05 V, and the device area of 0.16 cm^2^ was defined using a black metal aperture. The external quantum efficiency (EQE) measurements were carried out using monochromatic illumination (300 W xenon arc lamp through Nikon G250 monochromator, Tokyo, Japan). Transient absorption (TA) measurements were carried out using a femtosecond (fs) TA setup. The pump and probe pulses were delivered by a titanium/sapphire laser (CPA-2010, Clark-MXR Inc., Dexter, MI, USA) with a pulse width of 150 fs, a wavelength of 775 nm, and a repetition rate of 1000 HZ. The laser light was split into two parts; one part was incident on a sapphire plate to produce white light for the probe beam. The other part was used as a pump pulse (wavelength can be tuned from 290 nm to 3 μm) converted from an optical parametric amplifier (a TOAPS from Quantronix, Hamden, CT, USA). The pump light was used to excite the sample. A silicon photodiode was exploited to collect the probe light passed through the sample. In this work, a pump light with a wavelength of 470 nm was used to excite all the samples. The intensity of pump light varied from 119 μJ/cm^2^ to 1.5 μJ/cm^2^.

## 3. Results and Discussion

**Figure 1:**
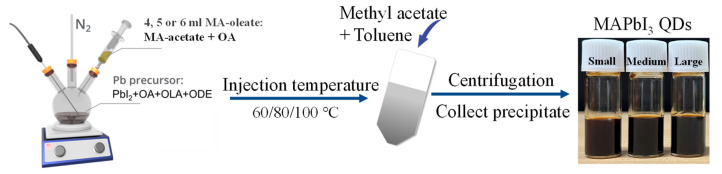
The schematic diagram of synthesizing MAPbI_3_ quantum dots.

**Figure 2:**
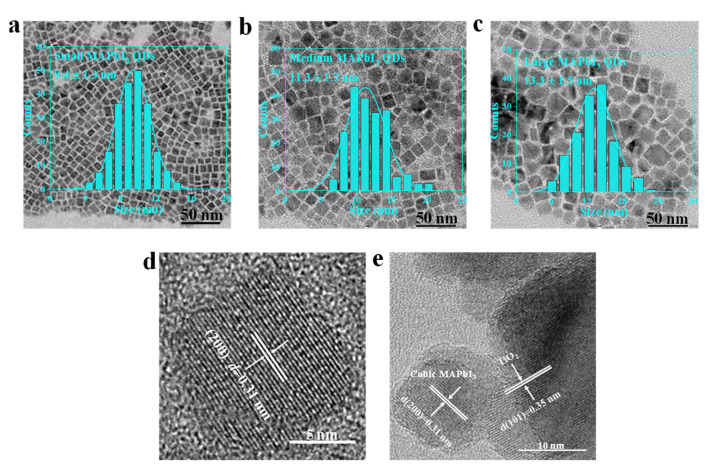
Transmission electron microscopy (TEM) images and corresponding size distribution histograms of (**a**) small, (**b**) medium, and (**c**) large size of MAPbI_3_ QDs. (**d**) High-resolution TEM pattern of the large MAPbI_3_ QD. (**e**) High-resolution TEM image of QDs/TiO_2_ composite.

Various sizes of MAPbI_3_ QDs were synthesized by a modified hot-injection approach (details can be found in Section 2), as illustrated in Figure 1 [30]. Figure 2a–c shows the representative transmission electron microscopy (TEM) images and their corresponding size distribution histogram of MAPbI_3_ QDs of three sizes: all QDs are nearly cubic shapes. The average size of small (S), medium (M) and large (L) MAPbI_3_ QDs are 9.4 ± 1.3, 11.3 ± 1.7 and 13.3 ± 1.5 nm, respectively, which are bigger than the exciton Bohr diameter of MAPbI_3_ (~5.6 nm) [31]; thus these QDs are in the weaker confinement regime. A high-resolution transmission electron microscopy (HRTEM) pattern of L-MAPbI_3_ QDs (Figure 2d) shows a lattice plane spacing of 0.31 nm corresponding to the (200) plane of the cubic MAPbI_3_ QDs [32]. The clear lattice fringes indicate the high crystallinity of the MAPbI_3_ QDs.

**Figure 3:**
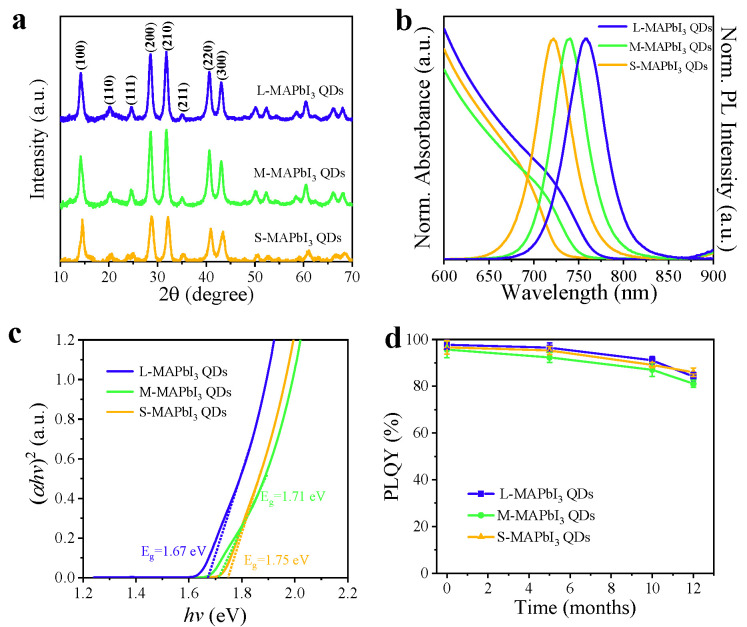
(**a**) X-ray diffraction patterns of various sizes of MAPbI_3_ QDs. (**b**) Normalized UV-visible absorption and steady-state photoluminescence spectra of various sizes of MAPbI_3_ QDs. (**c**) Tauc plot of the optical band gap energy for various size of MAPbI_3_ QDs calculated by extrapolation of the linear part of (*αhv*)^2^ versus (*hv*). (**d**) The evolution of PLQY for various size of MAPbI_3_ QD colloidal solution saved in ambient conditions.

X-ray diffraction (XRD) measurement was performed to further identify the crystallinity of obtained MAPbI_3_ QDs, as presented in Figure 3a, and all three sizes of MAPbI_3_ QDs showed a pure cubic phase [33,34]. The typical diffraction peaks at 2θ with 14.10°, 28.51°, 31.75°, 40.68° and 43.16° corresponded to (100), (200), (210), (220) and (300), respectively. Furthermore, both the normalized absorption and steady-state photoluminescence (PL) spectra of MAPbI_3_ QDs in hexane with different sizes exhibited continuous tunability, as can be seen in Figure 3b. To determine the optical band gap energy of various sizes of MAPbI_3_ QDs, the dependency of (*αhv*)^2^ upon the incident photons energy *hv* was plotted, as depicted in Figure 3c, and the band gap (*E_g_*) was estimated by extrapolating the linear part of (*αhv*)^2^ versus (*hv*) [35]. As shown in Table 1, the *E*_g_ of small, medium, and large MAPbI_3_ QDs was 1.75 eV, 1.71 eV, and 1.67 eV, correspondingly. Analogously, the PL peak positions of various sizes of QDs were tuned from 722 to 758 nm. As-prepared MAPbI_3_ QDs display a high PLQY of 97.8 ± 1.9%, 95.7 ± 3.5% and 96.6 ± 2.9% for large, medium, and small QDs (Table 1), respectively; after being saved in ambient conditions for 12 months, they still exhibited 84.2 ± 1.7%, 81.1 ± 1.5%, and 86.0 ± 1.8% (Figure 3d), correspondingly, suggesting the high quality of these QDs. The adsorption of QDs on the Al_2_O_3_ or TiO_2_ substrate was carried out by immersing the films in a hexane colloidal solution of MAPbI_3_ QDs for 8 h before rinsed using hexane and drying with nitrogen (details are provided in Section 2.3) [19,23]. The HRTEM in Figure 2e also confirmed that MAPbI_3_ QDs had been successfully and chemically adsorbed on TiO_2_ (lattice plane spacing of 0.31 and 0.35 nm is associated with the (200) and (101) plane of cubic MAPbI_3_ QDs and TiO_2_, respectively). This monolayer adsorption can ensure that the charge transfer dynamics at the interface of QDs and CTL can be studied without other complicated processes.

**Figure 4:**
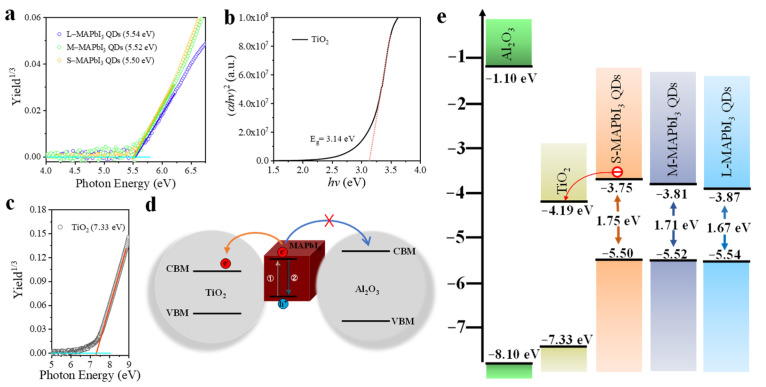
(**a**) PYS spectra of various size of MAPbI_3_ QDs. The valence band energy level of measured QD sample was obtained from the extrapolation of the linear portion in the spectrum. (**b**) Tauc plot of the optical band gap energy for mesoporous TiO_2_ film calculated by extrapolation of the linear part of (*αhv*)^2^ versus (*hv*). (**c**) PYS spectrum of mesoporous TiO_2_ film. (**d**) Schematic energy level diagram of the effective transfer pathway of photogenerated carrier in MAPbI_3_ QDs adsorbed on TiO_2_ and Al_2_O_3_ films, CBM is conduction band minimum, VBM represents valence band maximum. (**e**) Diagram of relative energy levels of MAPbI_3_ QDs of three kinds of size, TiO_2_ and Al_2_O_3_.

To confirm the thermodynamical feasibility of charge transfer from all MAPbI_3_ QDs to TiO_2_, firstly, the photoelectron yield spectra (PYS) of all QDs were measured, and the valence band energy level of each QD sample was obtained from the extrapolation of the linear portion of each spectrum, as shown in Figure 4a. The band gap of all QDs was determined from the Tauc plots, as discussed above. For the TiO_2_, similarly, the band gap and position of the valence band maximum (VBM) were determined by the Tauc plot (Figure 4b) and PYS spectra (Figure 4c), respectively. All sizes of MAPbI_3_ QDs showed a shallow conduction band (CB) energy level (−3.87, −3.81, −3.75 eV for large, medium and small QDs, respectively) compared to that of TiO_2_ (−4.19 eV), which were in favor of the charge transfer, while the Al_2_O_3_ acted as a charge-transfer barrier for its high CB edge (electron injection is not thermodynamically feasible) [19,36,37]. Figure 4d–e schematically exhibits the available charge transfer pathway in MAPbI_3_ QDs. Therefore, a comparison of the charge transfer dynamics of the QD solution, adsorbed on Al_2_O_3_ as well as TiO_2_ films, allows us to estimate the transfer rate of the carrier from QDs to TiO_2_.

**Figure 5:**
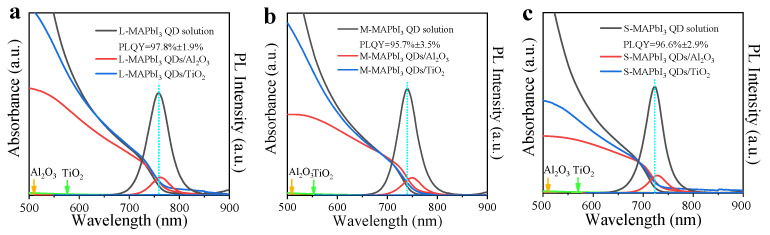
The absorption and PL spectra of (**a**) L-MAPbI_3_ QD solution, L-MAPbI_3_ QDs/TiO_2_ and L-MAPbI_3_ QDs/Al_2_O_3_, (**b**) M-MAPbI_3_ QD solution, M-MAPbI_3_ QDs/TiO_2_ and M-MAPbI_3_ QDs/Al_2_O_3_, (**c**) S-MAPbI_3_ QD solution, S-MAPbI_3_ QDs/TiO_2_ and S-MAPbI_3_ QDs/Al_2_O_3_.

In particular, as shown in Figure 5a–c, an obvious PL quenching of MAPbI_3_ QDs adsorbed on insulating Al_2_O_3_ films was observed. Considering that there was no charge transfer between QDs and Al_2_O_3_, this quenched PL may result from the absence of surface ligands of QDs attached to Al_2_O_3_ films, which can lead to the introduction of nonradiative recombination sites. As expected, the MAPbI_3_ QDs adsorbed on TiO_2_ shows great PL quenching compared to the QD solution and that attached on Al_2_O_3_ films (Figure 5a–c), suggesting that the effective charge transfer occurred in QDs/TiO_2_ heterojunctions [19,23].

**Figure 6:**
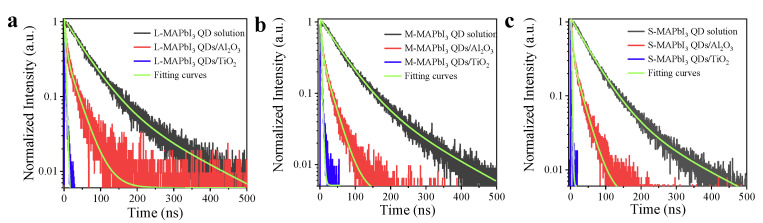
Time-resolved photoluminescence (TRPL) decay of (**a**) large, (**b**) medium, (**c**) small MAPbI_3_ QD solution, adsorbed on TiO_2_ and Al_2_O_3_.

In addition, time-resolved photoluminescence (TRPL) measurements were employed to examine the carrier kinetics of various sizes of MAPbI_3_ QDs and QDs/MOs heterojunction, as shown in Figure 6a–c. All the decay curves of various sizes of QDs as well as QDs/Al_2_O_3_ were well fitted by a biexponential model (*y* = *A*_1_*exp*(−*t*/*τ*_1_) + *A*_2_ *exp*(−*t*/*τ*_2_), where *A*_1_ and *A*_2_ are constant, *τ*_1_ and *τ*_2_ represent lifetime, and *t* is time), and the obtained parameters were listed in Table 2. Since there was nearly a 100% PLQY of all sizes of MAPbI_3_ QDs mentioned above, the nonradiative recombination process of the carrier could be neglected; thus, the fast process (*τ*_1_, approximately 50 ns) with dominant contribution (>90%) of all three sized QDs could be ascribed to the radiative recombination of excitons (electron–hole pairs), while the slower process may have resulted from the quasi-free carrier radiative recombination, due to the weaker quantum confinement effect of these QDs. (The photoexcited excitons of QDs can rapidly dissociate to become quasi-free carriers, which then decay through bimolecular recombination) [19,38]. The average lifetimes were calculated by $\tau_{ave}=\frac{\sum A_{i}\tau_{i}^{2}}{\sum A_{i}\tau_{i}}$, and those of all QD solution were similar (68~80 ns). In particular, it was found that the QDs attached to Al_2_O_3_ showed a shorter average lifetime (18~23 ns) than those of the QD solution, which was caused by the introduced defects. In addition, the decay curves of QDs/TiO_2_ heterojunctions were fitted using a mono-exponential equation (Table 2). As can be seen from Figure 6a–c, the QDs attached on TiO_2_ films present a much shorter PL lifetime (<2.8 ns) than those adsorbed on Al_2_O_3_ as well as the QD solution, which gives important evidence that the effective charge transfer occurred in this heterojunction and indicates that the charge transfer process was the dominant route of the photoexcited carrier in QDs/TiO_2_.

**Figure 7:**
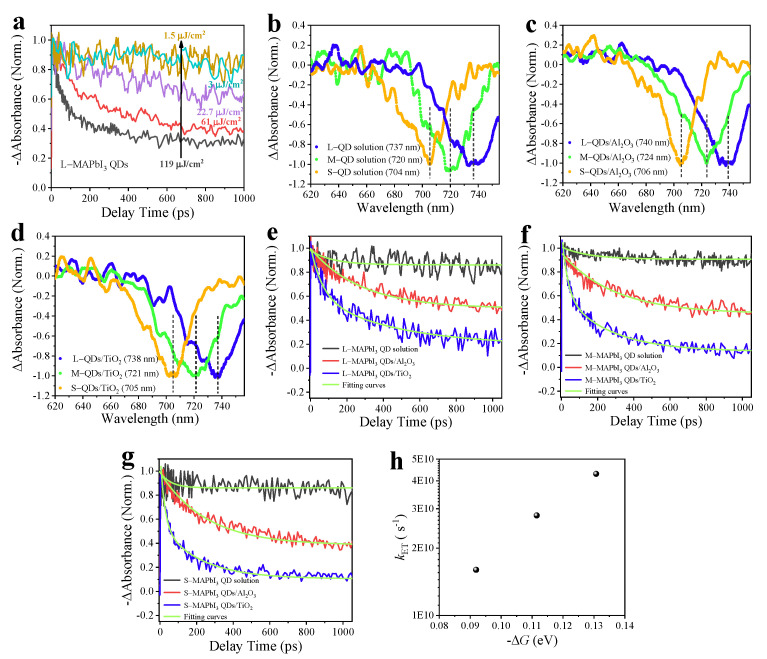
(**a**) The transient absorption (TA) kinetics of large size of MAPbI_3_ QDs under different excitation intensities (1.5~119 μJ/cm^2^), fast decay processes of TA responses appear when the excitation intensity is larger than 3.0 μJ/cm^2^, and become significant as the intensity increases. TA spectra of various sizes of (**b**) MAPbI_3_ QDs dispersed in hexane, (**c**) QDs/Al_2_O_3_, (**d**) QDs/TiO_2_ recorded at 5 ps and excited at 470 nm with an excitation intensity of 1.5 μJ/cm^2^. TA decay curves of (**e**) large, (**f**) medium and (**g**) small QD colloidal solution, attached on TiO_2_ and Al_2_O_3_. (**h**) Charge transfer rate constant (*k*_CT_) of MAPbI_3_ QDs as a function of the free energy change (−Δ*G*).

Nevertheless, the nanosecond timescale is difficult when revealing charge transfer dynamics in the QDs/TiO_2_ heterojunction. Thus, femtosecond transient absorption (TA) measurements were carried out at an excitation wavelength of 470 nm. The three-body Auger recombination process may appear at a higher excitation intensity, and this process generally is much faster than the bimolecular recombination, thus, can possibly overlap with the charge transfer process, which makes the discussion complicated [39]. First, the excitation intensity was adjusted from 119 to 1.5 μJ/cm^2^ to avoid the fast Auger recombination. As shown in Figure 7a, the fast decay component appeared in TA responses when the excitation intensity was larger than 3.0 μJ/cm^2^, and became more significant with the increase in intensity. When the excitation intensity was adjusted to 3.0 μJ/cm^2^, the fast decay process disappeared, and the normalized TA decay curves overlapped as well, which suggested that the Auger recombination was eliminated. The TA spectra of all sizes of QDs in hexane, QDs attached on the Al_2_O_3_ and TiO_2_ were collected 5 ps after band gap excitation under 1.5 μJ/cm^2^ and are exhibited in Figure 7b–d. The wavelength of each bleaching maximum of TA spectra coincides with that of the lowest excitation state transition of absorption spectra (Table 1). All TA kinetic curves of QD solution and QDs/Al_2_O_3_ monitored at each bleaching maximum can be well fitted by the single-exponential function (Figure 7e–g): *y* = *A*_0_ exp (−*t*/*τ*) + *y*_0_. As shown in Table 3, the fitting lifetimes of the large, medium, and small MAPbI_3_ QD colloidal solution are 123, 150 and 134 ps, respectively. Considering the absence of Auger recombination, these lifetimes can be assigned to the nonradiative combination (i.e., carrier trapping in defect states), although this process has been ignored in the nanoscale (the results in TRPL characterization). The constant component (lifetime >> 1 ns) can be attributed to radiative recombination. For the TA decay of QDs/Al_2_O_3_, the fitted lifetimes (278, 291, and 253 ps for large, medium, and small QDs/Al_2_O_3_, respectively) with an amplitude from 51% to 60% were considered to originate from the nonradiative recombination, whereas the constant part may mostly reflect the radiative recombination processes. In the case of QDs/TiO_2_, the TA kinetics of all sized QDs/TiO_2_ were determined to be biexponential (*y* = *A*_1_*exp*(−*t*/*τ*_1_) + *A*_2_ *exp*(−*t*/*τ*_2_) + *y*_0_) (Figure 7e–g), as shown in Table 3, an additional faster decay of the signal traces with the lifetime of 62, 36, and 23 ps for large, medium, and small QDs, separately, was obtained. The fitting of the later slower parts of decay curves with a time constant of 215~331 ps was close to those of the QDs/Al_2_O_3_, which could be assigned to the nonradiative recombination. Thus, the fast parts of kinetics could be attributed to the charge transfer process from QDs to TiO_2_. The rate of charge transfer (*k*_CT_) from QDs to TiO_2_ could be calculated from: 1/*τ*_1_, those of large, medium, and small QDs were 1.6 × 10^10^, 2.8 × 10^10^and 4.3 × 10^10^ s^−1^, correspondingly. It is worth noting that the *k*_CT_ of MAPbI_3_ QDs depends on the *QD* size. This was perhaps caused by the different free energy forces (−Δ*G*) of these QDs/TiO_2_ systems. According to the Marcus theory, the *k*_CT_ is a function of Δ*G*, which is the free energy change between the donor and acceptor and associated with the QDs size [40,41]. We calculated the Δ*G* through the following Equations:

(1) $$\Delta G=\Delta E_{electron}+\frac{e^{2}}{2R_{QD}}+\frac{2.179e^{2}}{\varepsilon_{QD}R_{QD}}-\frac{e^{2}}{4\left(R_{QD}+h\right)}\frac{\varepsilon_{acceptor}-1}{\varepsilon_{acceptor}+1}$$

(2) $$\Delta E_{electron}=CBM_{acceptor}-CBM_{QD}$$

where Δ*E_electron_* is the energy difference between the conduction band edge of the acceptor semiconductor TiO_2_ and QDs, *e* is the elementary charge, *R_QD_* represents the radius of QDs, *h* is the distance between QDs and TiO_2_ (the radius of QDs is very large compared to *h*; thus, it can be set to 0), and the *ε_acceptor_* and *ε_QD_* are the dielectric permittivity of TiO_2_ and QDs (*ε*_TiO2_ = 80, *ε_QD_* = 26) [42,43]. We obtained the Δ*G* value using Equations (1) and (2), as shown in Figure 7h, and the value was −0.09, −0.11 and −0.13 eV for small, medium, and large QDs.

**Figure 8:**
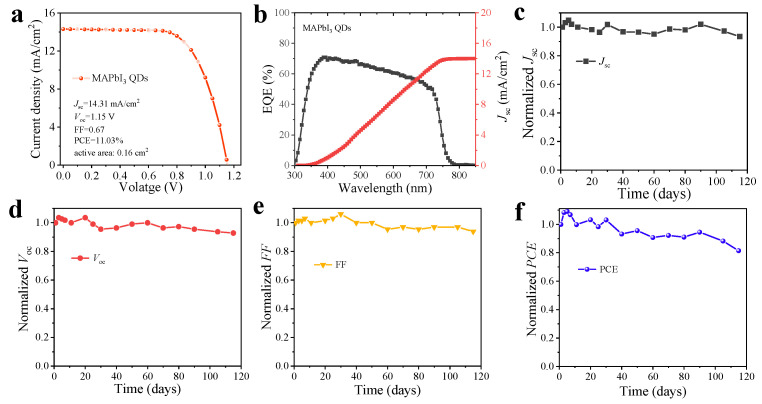
(**a**) The *J–V* curve and (**b**) corresponding EQE of MAPbI_3_ QDs/TiO_2_-based solar cell. Evolution of the normalized (**c**) short-circuit current density (*J*_sc_), (**d**) open-circuit voltage (*V*_oc_), (**e**) fill factor (*FF*) and (**f**) power conversion efficiency (*PCE*) of QD solar cell, which was kept in a dry cabinet (<30% humidity) and measured in ambient air.

Finally, we successfully fabricated the QD solar cell with the structure of glass/FTO/TiO_2_/QDs/spiro-OMeTAD/Au with the active area of 0.16 cm^2^ (the details of the fabrication can be found in Section 2.4) using the medium size of QDs. The representative *J–V* curve is presented in Figure 8a, and the MAPbI_3_ QDs/TiO_2_-based solar cell achieved a high PCE of 11.03%, with a *J*_sc_ of 14.31 mA/cm^2^, *V*_oc_ of 1.15 V, and fill factor (FF) of 67%, which demonstrated a significant enhancement over the same composition QD solar cells [21]. Figure 8b shows the external quantum efficiency (EQE) spectrum of this device, which is in good agreement with the *J*_sc_ value obtained from the *J–V* curve. Additionally, the MAPbI_3_ QD-based solar cell device exhibited high stability, which could maintain ~81% of its initial PCE after 115 days of storage under an atmosphere with a humidity of <30% (Figure 8c–f). According to the previous experimental and theoretical studies, the efficiency of perovskite solar cells could be significantly improved through the application of metallic nanoparticles in the perovskite layer, or at the interface between the charge transfer layer and absorber layer due to the optical plasmon photovoltaic effect and intrinsic electric plasmonic effects [44,45]. Furthermore, the binding energy of the excitons could be reduced through the plasmons of metallic nanoparticles and the rapid dissociation of excitons that took place at the interface of the charge (electron or hole) transport layer and perovskite layer, which could significantly increase the photo-current of perovskite solar cells [45]. For PQD solar cells, low carrier mobility is the main reason for such lower *J*_sc_ than that of bulk perovskite solar cells. Hence, the PQD solution dopped with metallic nanoparticles and the interface between the PQD layer and charge transport layer modified by metallic nanoparticles produced promising approaches for increasing the photocurrent of PQD solar cells and further optimizing the efficiency of PQD solar cell devices.

## 4. Conclusions

In summary, the charge transfer dynamics of MAPbI_3_ QD/MOs were comprehensively investigated using a TA characterization method. Effective charge injection in MAPbI_3_ QD/TiO_2_ heterojunctions was observed, and the size dependence of the rate of the charge transfer from QDs to TiO_2_ was further verified in our work. The rate of the charge transfer from large (13.3 ± 1.5 nm), medium (11.3 ± 1.7 nm), and small (9.4 ± 1.3 nm) QDs to TiO_2_ were determined to be 1.6 × 10^10^, 2.8 × 10^10^, and 4.3 × 10^10^ s^−1^, respectively. Furthermore, a high PCE of 11.03% based on QDs/TiO_2_ junctions was also achieved, demonstrating the great potential of using MAPbI_3_ QDs in high-performance photovoltaic devices.

## Figures and Tables

**Table 1:** The determined band gap, PL peak position and corresponding PLQY of three-sized MAPbI_3_ QDs.

MAPbI_3_ QDs	*E_g_* (eV)	*λ*_PL_ (nm)	PLQY (%)
L-QD solution	1.67	758	97.8 ± 1.9
M-QD solution	1.71	740	95.7 ± 3.5
S-QD solution	1.75	722	96.6 ± 2.9

**Table 2:** The fitted parameters of TRPL decay profiles for various sizes of MAPbI_3_ QD solution, QDs/TiO_2_ and QDs/Al_2_O_3_. The decay curves were fitted by a biexponential (*y* = *A*_1_*exp*(−*t*/*τ*_1_) + *A*_2_ *exp*(−*t*/*τ*_2_)) and single-exponential model (*y* = *A*_1_ exp (−*t*/*τ_1_*)), where *A*_1_ and *A*_2_ are constant, *τ*_1_ and *τ*_2_ represent lifetime, *t* is time. *τ*_ave_ is average lifetime calculated by $\tau_{ave}=\frac{\sum A_{i}\tau_{i}^{2}}{\sum A_{i}\tau_{i}}$.

MAPbI_3_ QDs	*A*_1_ (%)	*τ*_1_ (ns)	*A*_2_ (%)	*τ*_2_ (ns)	*τ*_ave_ (ns)
L-QD solution	92.0	50.8	8.0	177.0	80.1
L-QDs/Al_2_O_3_	67.2	4.2	32.8	28.7	23.0
L-QD/TiO_2_	100	2.8	–	–	2.8
M-QD solution	93.5	51.9	6.5	163.3	71.9
M-QDs/Al_2_O_3_	73.7	4.0	26.3	24.9	18.4
M-QDs/TiO_2_	100	2.4	–	–	2.4
S-QD solution	93.7	48.5	6.3	159.7	68.7
S-QDs/Al_2_O_3_	70.6	4.3	29.4	25.1	19.0
S-QDs/TiO_2_	100	1.4	–	–	1.4

**Table 3:** The fitted parameters of TA kinetics for various size of MAPbI_3_ QD colloidal solution, QDs/TiO_2_ and QDs/Al_2_O_3_. The decay curves were fitted by single-exponential (*y* = *y*_0_ + *A*_1_ *exp*(−*t*/*τ*_1_)) or biexponential model (*y* = *A*_1_ *exp*(−*t*/*τ*_1_) + *A*_2_ *exp*(−*t*/*τ*_2_) + *y*_0_), where *y*_0_, *A*_1_, *A*_2_ are constant, *τ*_1_ and *τ*_2_ represent lifetime, *t* is time.

MAPbI_3_ QDs	*A*_1_	*τ*_1_ (ps)	*A*_2_	*τ*_2_ (ps)	*y*_0_	*k*_CT_ (s^−1^)
L-QD solution	0.08	123	–	–	0.92	–
L-QDs/Al_2_O_3_	0.51	278	–	–	0.49	–
L-QD/TiO_2_	0.40	62	0.41	331	0.19	1.6 × 10^10^
M-QD solution	0.09	150	–	–	0.91	–
M-QDs/Al_2_O_3_	0.55	291	–	–	0.45	–
M-QDs/TiO_2_	0.49	36	0.40	287	0.11	2.8 × 10^10^
S-QD solution	0.09	134	–	–	0.91	–
S-QDs/Al_2_O_3_	0.60	253	–	–	0.40	–
S-QDs/TiO_2_	0.53	23	0.38	215	0.09	4.3 × 10^10^

## Data Availability

The data that support the findings of this study are available from the corresponding author upon reasonable request.
